# Does Placement of Timing Sensors and Sprinting Model Alter Force–Velocity Metrics? A GNSS Simulation Study

**DOI:** 10.3390/s26144642

**Published:** 2026-07-22

**Authors:** Ming-Chang Tsai, Daniel Geneau, Dana Agar-Newman, Isobel Chaudhry, Patrick Cormier, Marc Klimstra

**Affiliations:** 1 Canadian Sport Institute Pacific, Victoria, BC V9E 2C5, Canada; 2 School of Exercise Science, Physical and Health Education, University of Victoria, Victoria, BC V8P 5C2, Canada

**Keywords:** optimization, global positioning system, sprint modelling

## Abstract

Horizontal force–velocity profiling (FV) has become a prominent approach to evaluate sprinting performance and force/power characteristics in athletes. As optical timing gates (TGs) are the most prevalent and accessible athlete sprint testing measurement devices, initial FV applications involved measuring sprint performance using TGs placed at the 0.75, 10, 15, 20, 30, and 40 m marks during all-out sprint testing sessions. However, there was uncertainty as to the standardization in the number, placement, and total distance of TG measurements to determine FV outputs. While original investigations have attempted to determine optimal TG measurement configurations, they have not been compared to continuous velocity measurements, nor have they been subject to a statistical optimization approach. Therefore, the objective of this study is to use simulated TG locations, derived from continuous GNSS velocity measurements, to identify the shortest distance with the minimum number of optimal timing-gate locations to obtain accurate estimates of FV metrics. A total of 49 elite women’s rugby sevens athletes performed 40 m sprint tests between 2015 and 2020. Data were collected using GNSS units (STATSports, Ireland), with athlete split times extracted every 2.5 m interval for the first 10 m, and every 5 m for the remainder of the sprint to simulate timing gates. FV metrics and times generated for all the combinations were compared to the standard model and GNSS models, respectively, using a one-way repeated measures analysis of variance (ANOVA) model. The shortest distance with an average percentage difference 0.27% (RMSE 1.43) for the standard model and 0.43% (RMSE 0.08) for the GNSS models were [0.75, 10-15-25] and [0.75, 5-10-30] combinations, respectively, across all FV measures. For individual FV metrics, different combinations can provide better estimations, indicating that certain regions of the sprint are required for the determination of specific metrics. Within this cohort, the best TG combinations for linear sprint testing are 10 to 15 m shorter than the current suggested combination, using three fewer gates. Further, alternative combinations may offer greater accuracy for specific FV metrics. This work provides an approach for the determination of the most accurate TG locations for teams with constraints on time, money, and equipment.

## 1. Introduction

In sports that are heavily dependent on maximal running efforts, such as soccer [1], football, rugby [2], and Australian Rules Football [3], sprint testing is a common practice to evaluate and monitor athlete performance. Additionally, sprint testing is often included as part of talent identification testing programs (RBC Training Ground, Australia eTID, G, etc.) for sports associated with high sprint speed. Horizontal force–velocity (FV) profiling has become a popular technique used to estimate athlete horizontal force and power characteristics throughout a linear sprint [4,5]. FV profiling has a large scope of application, including strength and conditioning programming, athlete monitoring, and rehabilitation design [6,7,8,9]. The standard FV approach [4] utilizes a mono-exponential velocity–time fitted model applied to linear sprint trials to derive horizontal acceleration, force, and power. This model requires continuous velocity data measured from sensors such as radar or laser. Another mathematically identical and popular approach is to use a position-time fitting Lambert function and data from optical timing gates (TG) [10]. This approach is similar to the velocity–time model but enables the use of TG sensors which are a more common athlete sprint testing modality in many daily training environments.

As the popularity of FV profiling increases, the methodology and practical applications continue to expand. Recent investigations elaborate on a foundational study by Samozino et al. [4], validating the use of different sensing technologies such as global navigation satellite systems (GNSS) and comparing them to original approaches [11]. For example, Cormier et al. [11] compared FV profiles developed using GNSS and TG sensors to radar. The results of this study demonstrated that while TG–FV comparisons provided suitable estimation of speed and power values, the estimated force variables provided low association and poor limits of agreement (LOA) to the standard radar measurement [11]. This is somewhat in contrast with Fornasier-Santos [12] who demonstrated greater association and better LOA for TG only when using a video correction at the start of the sprint. Regardless, both studies demonstrated that the use of models with TG data results in overestimations in important FV acceleration-based metrics (e.g., F_0_), possibly due to the low sampling resolution toward the beginning of the sprint, where the change in velocity (linear acceleration), and horizontal force application is the highest. While the use of TG sensors to acquire sprint FV metrics may require technical improvements, they are still the most prevalent sensors used to measure athlete sprint performance as they are often the most readily available and user-friendly option. As there is growing interest in the use of FV to monitor performance at many levels, there is great value in determining the most appropriate methodological approach to TG–FV set-up and analysis. For example, the optimal configuration regarding the number and location of TGs to adequately measure athlete velocity has yet to be established.

While TG sensor use for FV profiling is common, there are often discrepancies between the exact configuration and location used to determine athlete sprint and ultimately develop individual FV profiles. Haugen et al. [13] used a [0.5 m, 10 m, 20 m, 30 m, 40 m] TG configuration, straying from the original configuration of [0 m, 10 m, 15 m, 20 m, 30 m, 40 m] [4]. This shows not only different placement of TG sensors but reducing the number of total split times present in the analysis. Other work by Helland et al. [14] used the same number of splits as Samozino et al.’s original configuration, but reduced the total sprint distance, with a configuration of [0 m, 5 m, 10 m, 15 m, 20 m, 30 m]. To address this lack of standardization, other work by Haugen et al. [15] explored several different TG locations. However, within this investigation, only 12 different gate combinations were compared, and no physical or statistical rationale was provided regarding the selection of those specific configurations. Therefore, a more systematic approach to identifying the optimal location for TG sensors has yet to be explored. The present state of TG use for FV profiling has revealed important gaps that require further investigation. First, there is a lack of validation of FV profile metrics for newly introduced measurement combinations. Second, there is uncertainty as to the optimal location, number of TGs, and sprint distance for valid and reliable measurement to support FV profiling. Further, there is uncertainty as to whether the optimal TG number, locations, and sprint distance are dependent on the athlete cohort for reliable and valid development of FV metrics. For example, as athletes with higher maximum velocity may achieve that higher velocity at a greater distance, this may impact the total sprint distance and may also alter the location of timing gates in reference to one another. More investigation into these considerations may reveal the optimal methodological approach for the use of TG sensors in accurately calculating FV profile metrics from maximal sprint efforts.

One approach to determining the optimal number and location of TGs, and sprint distance, for valid and reliable development of an FV metric is to simulate multiple permutations of the number and locations of TGs from a continuous velocity measurement, such as that obtained from video, radar, laser, or GNSS sensors. A promising source of continuous velocity data is from athlete-worn GNSS sensors, which have proven valuable at collecting sprint data from many athletes within a cohort and have often replaced radar or laser as a preferred method [8,11,16]. While TGs are more often used at developmental athlete levels, GNSS sensors are commonly used for more elite athlete levels to monitor in-game or training kinematics of athletes, including athlete speed, acceleration, and displacement [2,17,18,19]. With GNSS devices collecting velocity data at 10–25 Hz [18,19,20] using the doppler technique, they have been shown to reliably develop FV profiles in team-sport athletes [11]. In a recent meta-analysis, ref. [8], it was found that valid FV profiles could be generated from GNSS technology when compared to standard radar/laser measurements (small standardized mean differences, <2 percent difference). GNSS technology can therefore provide the continuous data necessary to simulate multiple TG locations throughout the sprint trial.

Therefore, the objective of this study is to identify the optimal TG number and location, as well as the shortest distance to obtain accurate estimates of FV metrics (i.e., the ‘best’ timing-gate configuration). This will be obtained by comparing FV metrics from multiple TG number and location iterations to the standard TG configuration, as well as metrics obtained using the continuous velocity signal derived from GNSS. Further, we aim to identify combinations that can best predict individual FV output variables. We hypothesize that some combinations with shorter overall distance and fewer split times will still reliably generate all FV measures. Finally, we also hypothesize that certain combinations may lend themselves to accurately estimating some FV measures while inaccurately determining others, essentially identifying regions of the sprint that must be measured for specific FV metrics. This study is therefore an optimization exercise, identifying TG configurations that best replicate FV outputs from a standard protocol and continuous signal, rather than a validation of TG-derived FV metrics against an independent, physically measured criterion.

## 2. Materials and Methods

### 2.1. Participants

A total of 49 highly trained/national-level-to-world-class (Tier 3–5) [21] women’s rugby sevens athletes from a national training program (height = 1.69 ± 0.60 m, mass = 70.70 ± 6.17 kg, age = 24.20 ± 4.44 years) performed 428 sprint-running tests (40 m) between 2015 and 2020. All athletes were non-injured at the time of collection. Ethical approval was obtained from the University of Victoria Human Research Ethics Committee and was conducted in accordance with the Declaration of Helsinki.

### 2.2. Testing Protocol

Athletes began each sprint in a 2-point stance and were instructed to maximally accelerate and run as quickly as possible past a measured 40 m mark. All sprints were conducted on artificial turf (Field Turf, Calhoun, GA, USA) in cleats. Data were collected using Apex 10 Hz GNSS (STATSports, Newry, North Ireland), positioned between the scapulae in the manufacturer-provided garment. Technical specifications of the units are presented in Table 1. The Apex 10 Hz contains an augmented multi-GNSS receiver capable of simultaneously acquiring signals from GPS, GLONASS, Galileo, and BeiDou satellite systems [22]. Instantaneous horizontal velocity was obtained from the receiver’s doppler-shift solution, which estimates receiver velocity from changes in the carrier frequencies of the signals transmitted by the GNSS satellites [11]. Thus, the doppler method refers to the processing of received satellite signals and not to the transmission of a radar signal by the wearable unit. Output measures of distance (m), instantaneous velocity determined using the doppler method (m/s), and instantaneous acceleration (m/s^2^) were used in this analysis. Sprint onset was determined as the first instance from rest where the athlete acceleration was above 0.1 m/s^2^. The manufacturer does not publicly disclose the exact differentiation, filtering, or sensor-fusion procedures used to generate this acceleration output. Importantly, this exported acceleration signal was not used directly to calculate the FV metrics. From sprint onset, the first simulated gate was at 0.75 m, in line with other established timing-gate configurations [11]. Additional split times were extracted from the continuous GNSS distance–time record at every 5 m intervals from 5 to 40 m (i.e., 5, 10, 15, 20, 25, 30, 35, and 40 m), providing eight candidate locations. These locations, together with the fixed 0.75 m split, were used to generate 3718 simulated timing-gate configurations containing between three and eight split times in total. The configuration containing all eight split times was therefore one of the candidate configurations evaluated and was not treated as a reference condition. Each configuration was fitted using the position-time Lambert function, and the corresponding FV metrics (F_0_, V_0_, P_max_, D_RF_, and Est_40m_) were calculated based on the methods detailed by Samozino [4] in R (Version 4.4.1, Vienna, Austria). This resulted in a total of 1,591,304 sprint calculations (see Supplementary Materials) from 3718 unique measurement locations.

### 2.3. Data Analysis

The FV metrics generated from each simulated timing-gate configuration were compared separately with tow reference conditions. The first reference, TG_s_, consisted of FV metrics calculated from the standard 0.75, 10, 15, 20, 30, and 40 m timing-gate configuration described by Samozino et al. [4]. The second reference, V_cont_, consisted of FV metrics calculated from the complete continuous 10 = Hz GNSS velocity–time signal. For Vcont, the continuous velocity observations were fitted using the mono-exponential velocity-tie model described by Samozino et al. [4], from which the corresponding FV metrics were derived. Thus, V_cont_ represented the complete continuous GNSS velocity signal and was independent of the simulated time-gate configurations. Separate one-way repeated measures analysis of variance (ANOVAs) were used to compare the FV metrics from each simulated timing-gate configuration with the TG_s_ and V_cont_ reference measures. A significance threshold of *p* < 0.05 was used. Further, Mauchly’s test was used to evaluate the assumption of sphericity, with the Greenhouse–Geisser correction applied when this assumption was violated. All analyses were conducted in the R programming language (Version 4.4) using the packages Multcomp, nlme, tidyverse, rstatix, emmeans, and car. The Bonferroni post hoc procedure of multiple comparison with TG_s_ as reference was used to control for type I errors in making multiple comparisons, to determine the significant difference between the metrics determined at each combination and TG_s_ metrics. Investigation of the residual plot showed a random scatter of points and the normality plot showed that the residuals fall on a straight line, indicating that the normality assumption was appropriate for the FV metrics. Further, Shapiro–Wilk tests confirmed the visual inspection, displaying no evidence of violation of the normality assumption. Given the large sample size (*n* = 428), statistical significance can be observed for a small difference; however, these potential differences may not be theoretically or practically relevant. Therefore, the significant combinations were further screened for extreme differences between the metrics using the boxplot method [23].

Individual FV metrics were averaged across athletes for each TG combination (*n* = 3718). The best TG location combinations were identified by calculating percentage difference values for each metric, for all TG combinations. Metrics which exhibited ‘extreme difference’ (those identified through boxplot analysis to be extremely different from Samozino’s metrics) were removed from analysis. Percentage difference values indicated the magnitude of the difference between new combination metric values and those generated using the TG_s_ configuration- or V_cont_ (continuous velocity from GNSS sensors)-derived metrics. Measures based on the V_cont_ signal were calculated using the mono-exponential velocity–time model outlined in the work by Samozino et al. [4]. The smaller the percentage difference value, the smaller the difference between the two metrics, and the greater the accuracy. Due to this, we were able to identify the shortest combination with the fewest TGs from this lower quartile, and label this as the ‘best combination’. To identify the best combination if all FV metrics were of interest, a similar analysis was conducted where only configurations where all FV metrics did not exhibit ‘extreme difference’ were passed for analysis. We then repeated the process of identifying the shortest combination with the fewest TGs and named this as the ‘best’ combination across all metrics. Bland–Altman plots were generated to investigate the level of agreements between best combination estimates and the TG_s_ estimates. A range of agreement was defined as mean bias ±2 SD with 96% percentage of values within the limits. The limit of agreement was defined as 5% a priori as acceptable. Linear regressions were used to determine the linear relationship between all combination and FV metrics estimated from TG_s_. The regression coefficient was tested against 1 (instead of the default 0 in t-statistics) to show the deviation from identity. Finally, coefficient of determination (r^2^) values were also calculated to further evaluate the relationship of calculated measures to those derived from the benchmark TG_s_ configuration.

## 3. Results

**Figure 1:**
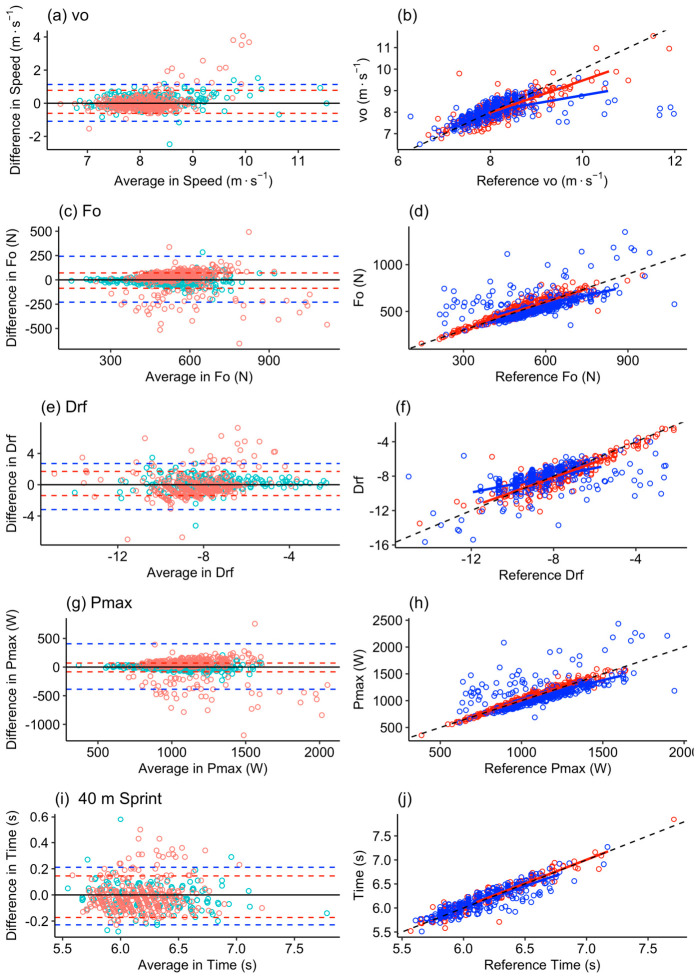
Linear regression and Bland–Altman analysis for the ‘best combination’ timing-gate configuration. The best combination was compared to the standard TG_s_ (red) and V_cont_ (blue) configuration for each FV profile outcome measure (panels (**a**–**j**), respectively).

A total of 3718 TG combinations were used to develop FV measures, with each combination compared to the TG_s_ and V_cont_ baseline measures. Descriptive statistics for TG_s_ and V_cont_ ANOVA analysis can be seen in Supplementary Table S1 and Table S2, respectively. It was observed that for both TG_s_ and V_cont_, a minimum of four gates were needed to accurately determine FV metrics. Combinations with fewer than four gates were removed from further analysis. To determine the ‘best combination’, only configurations where all FV metrics were determined as significantly non-different were considered. The ‘best combination’ was identified as the configuration with the smallest number of gates, shortest distance, and lowest overall percentage difference from comparative measures. For both TG_s_ and V_cont_ comparisons, the respective ‘best combinations’ are outlined in Table 2 and Table 3. Further, Figure 1 compares the best combinations for TG_s_ and V_cont_ benchmarks across all FV metrics using Bland–Altman and linear regression analysis.

**Figure 2:**
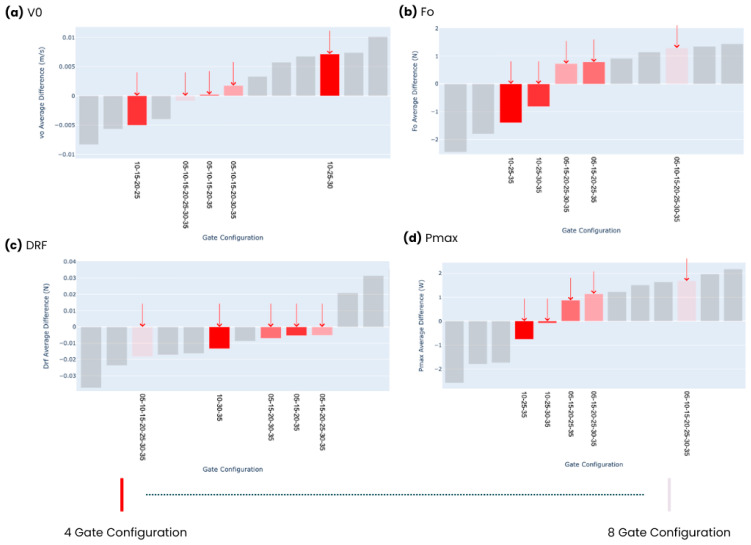
For each 4, 5, 6, 7, and 8-gate combination shorter than 40 m not significantly different from the TG_s_ configuration, the best combination of gate placements was identified for individual FV metrics as the smallest percentage difference from the TG_s_ baseline value. These ‘best’ configurations are highlighted in red for each metric (V_0_, F_0_, DRF, and Pmax) in subfigure (**a**), (**b**), (**c**), and (**d**), respectively.

**Figure 3:**
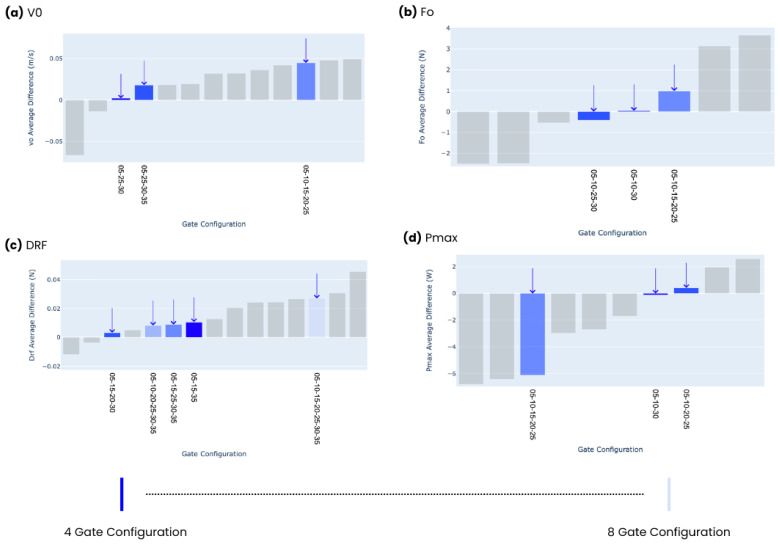
For each 4, 5, 6, 7, and 8-gate combination shorter than 40 m not significantly different from the V_cont_ configuration, the best combination of gate placements was identified for individual FV metrics as the smallest percentage difference from the V_cont_ baseline value. These ‘best’ configurations are highlighted in red for each metric (V_0_, F_0_, DRF, and Pmax in subfigure (**a**), (**b**), (**c**), and (**d**), respectively.

For the determination of ideal gate combinations for specific FV metrics, all combinations not different for individual FV metrics were included in the analysis. For each metric, combinations were compared to the TG and V_cont_ baseline measures, as seen in Figure 2 and Figure 3, respectively. The best combinations for individual metrics were then identified for each gate number grouping (4, 5, 6, and 7 gates). Here, the absolute average difference, RMSE, and percentage difference from baseline measures for TG_s_ and V_cont_ comparisons can also be seen in Table 4.

## 4. Discussion

This study investigated how the number and placement of timing gates (TGs) influence the accuracy of horizontal force–velocity (FV) profiling, using simulated TG splits derived from GNSS data and comparing them to both a standard gate configuration (TG_s_) and continuous velocity (V_cont_) measures. The findings highlight that both the number of timing gates and the total sprint distance can be reduced without compromising the accuracy of FV measures. Specifically, for both TG_s_ and V_cont_ based comparisons, the optimal configurations consisted of four timing gates distributed over shorter sprint distances (25 m and 30 m, respectively). These results have important practical implications, suggesting that FV profiling can be conducted with fewer sensors and reduced sprint distances, thereby lowering athlete testing load while improving accessibility in applied settings. Additionally, it was observed that different gate configurations could reliably detect individual FV measures independently, indicating that testing procedures may be able to be tailored to specifically target specific measures. Collectively, these findings provide a practical framework for optimizing timing-gate use in FV profiling, offering practitioners clear, context-specific guidance for implementation in training environments.

Previous studies have proposed a range of TG configurations for estimating FV metrics [2,14,24]. However, the only combination that has been validated against force plates is that of Samozino et al. [25], which utilized gates positioned at 0, 10, 15, 20, 30, and 40 m. In this validation, video analysis was used to correct for sprint onset. While this approach represents a robust research standard, it is often impractical in high-performance environments due to the time and resources required for video processing. As a result, simpler timing-gate–only approaches are generally preferred in applied settings. Since this initial validation, other work has explored alternative TG configurations for FV profiling, comparing 12 combinations to the validated protocol [15]. While fundamentally similar to the present investigation, the study by Haugen et al. had several limitations. First, the selection of timing-gate configurations was limited to 12 combinations without explicit rationale. Second, while intra-class correlation analysis was conducted to evaluate the agreement between configurations, there was no statistical comparison between gate combinations to determine if the outputs were significantly different to the baseline measure. As a result, the analysis was limited in its practical application, making it challenging to generalize the results and identify an optimal configuration for practitioners. This is particularly important, as shorter sprint distances are often required in applied sport environments due to space constraints and the need to manage athlete training load. Further, if practitioners are interested in individual FV metrics, gate configurations and required distances may vary. The systematic approach used in the present study to evaluate the validity of timing-gate configurations for FV analysis represents a substantial advancement over previous work.

In the present analysis, ‘best’ combinations using both TG_s_ and V_cont_ comparative measures were substantially shorter than the standard protocols and used only 3 split times (for a total of 4 gate sensor sets) with combinations of [0.75, 10, 15, 25] and [0.75, 5, 10, 30] for the TG_s_ and V_cont_ comparisons respectively. Differences between the results of these two methods can be explained by a few factors. In the initial validation of this technique by Samozino et al. [4] both continuous velocity from laser and optical timing-gate split segments were used to construct FV measures, with a marginal increase in error observed in the continuous velocity measure. However, in the more recent literature, continuous velocity data has consistently been used as the relative ‘gold standard’ measure for FV profiling as it provides both a direct velocity measure and higher resolution data [11,26,27]. Further, the split values used for the TG_s_ baseline were derived from the continuous GNSS signal and, as a result, introduced some level of measurement error. These factors may have led to the discrepancies seen in the results between methods. The rationale to include the TG_s_ comparative measure was for accessibility of this approach to practitioners in the field, understanding that many sport practitioners may not have access to equipment such as GNSS, radar, or laser. Therefore, to make this research as impactful and scalable as possible, both measures were included in the analysis.

When evaluating individual measures within the FV profile, specific regions proved to be more fundamental in the determination of specific characteristics. For example, the best fit for V_cont_-derived Pmax [0.75, 5, 10, 22] includes three gates within 10 m of the sprint, indicating that higher resolution is needed in this sprint phase to accurately model athlete power. Conversely, the best fit for TG_s_-derived DRF [0.75, 25, 30, 40] requires a larger sprint distance, with TG_s_ more distributed toward the end of the sprint. It was also apparent that some measures had substantial improvements in absolute error with the increase in splits. This is especially clear in the TG_s_ comparisons for F_0_ as seen in Table 4, where the error noticeably increases from the five-to-four-gate-combination fits. This trend is also shown in Figure 2, outlining the absolute error of each gate combination. It is notable that a similar trend is not as apparent in the V_cont_ comparative measure, indicating that there is perhaps a systematic bias for specific measures derived using the originally validated timing-gate configuration. It is likely that this bias is present in the F_0_ measure, as this subsequently is used to determine P_max_.

These findings highlight some of the biomechanical underpinnings of how the various FV measures are determined and the regions of the sprint effort that lend to the construction of each. Using the methodology outlined by Samozino et al. [4], FV measures F_0_ and, consequently, P_max_ are heavily reliant on the initial acceleration phase of the sprint effort, where horizontal force production and the forward orientation of the ground-reaction force are greatest [5] and where the velocity–time curve exhibits its steepest curvature. This mechanical pattern has also been demonstrated directly via force-plate analysis, with Rabita et al. [28] reporting that the ratio of horizontal-to-resultant ground-reaction force is highest during the earliest steps of the sprint and declines progressively as velocity rises. This is reflected in the results of the present study, with the best-fitting combinations for P_max_ and F_0_ requiring a heavier concentration of TG sensors earlier in the sprint effort. In contrast, V_0_ corresponds to the asymptotic portion of the velocity–time curve [4,7], where velocity changes slowly and is therefore well estimated even by widely spaced late-sprint samples. Further, DRF reflects the athlete’s ability to maintain effective horizontal force application as velocity rises—a technical quality that manifests progressively across the acceleration-to-maximal-velocity transition [5]—which explains why gate configurations distributed across a longer portion of the sprint, rather than concentrated early, more accurately captured this metric. Together, these patterns suggest that gate placement requirements are not arbitrary but map onto known phase-specific changes in force application during sprint acceleration, a relationship independently corroborated using force-plate methodology distinct from the timing-gate and GNSS approaches used in this and prior studies [28].

The intention of this work is to provide guidelines to practitioners using TG to assess FV profiles using maximal horizontal sprinting in daily training environments. However, one key limitation of the present study is the population used. The data used for this TG optimization was performed on a unique elite cohort of national-level female rugby athletes. The results therefore are specific to this population but could be supportive of developmental levels in elite women’s rugby where TGs may offer the most accessible sprint measurement and comparisons to an elite standard may be desired. Additionally, this study could be used an exemplar approach to determine the optimal TG number and locations for other elite-sport populations where sprint measurement and FV profiling is of interest. Further, the simulated timing-gate splits were indirectly derived from GNSS sensors rather than measured directly with optical TG hardware. While wearable GNSS sensors have been shown to be a reliable means of monitoring athlete speed and distance during sprint trials [11], GNSS-derived distance and velocity error is known to be largest during high-acceleration phases of the sprint [11,20], which may disproportionately affect early-sprint, acceleration-sensitive FV metrics such as F_0_. A direct comparison between simulated and physically-measured TG splits at the same locations, and against existing empirical TG configuration data such as Haugen et al. [15], represents an important next step to confirm that these findings generalize beyond the simulated context. An additional consideration is that the 428 sprints analyzed were not independent observations, as they were contributed by 49 athletes with an average of 12.20 ± 5.24 trials, with a minimum of two and a maximum of 20 trials per athlete. The repeated measures ANOVA comparing TG combinations to the standard configuration did not explicitly model this within-athlete clustering. This may violate the independence assumption underlying the analysis and could inflate the risk of Type I error. However, identification of the best-fitting TG combinations relied primarily on percentage difference (effect size) and boxplot-based screening for practical relevance, rather than statistical significance alone, which likely reduces the extent to which this clustering influenced the study’s core conclusions.

The present findings demonstrate that horizontal FV profiling protocols can be adapted to reduce equipment requirements, sprint distance, testing time, and athlete load while maintaining acceptable accuracy. However, the configurations identified are specific to national-level female rugby athletes and should not be assumed to generalize directly to other populations. Rather, the analytic approach used in this study provides a framework for developing context-specific timing-gate configurations based on the characteristics and practical constraints of a given sporting environment.

### Limitations and Future Directions

Several limitations should be considered when interpreting the present findings. First, all timing-gate splits were simulated from the GNSS distance–time record rather than measured directly using optical timing-gate hardware. This includes both the candidate timing-gate configurations and the standard 0.75, 10, 15, 20, 30, and 40 m configuration used as the primary timing-gate reference. Although this reference configuration was selected because it reflects the established method described by Samozino et al. [4], the split times used in the present analysis were simulated and should therefore not be interpreted as an independently measured ground truth.

The continuous-velocity reference was calculated from the complete GNSS velocity–time signal using the mono-exponential model described by Samozino et al. [4]. However, both the simulated timing-gate splits and the continuous-velocity reference originated from the same GNSS recording. Consequently, the comparison conditions may share common sources of measurement error, including GNSS-derived distance and velocity error, and are not statistically or technologically independent. This shared measurement source may increase the apparent agreement between the simulated timing-gate configurations and the reference measures. In particular, GNSS error is known to be greatest during high-acceleration phases of sprinting, which may disproportionately affect acceleration-sensitive outcomes such as F0.

Future studies should directly compare the identified configurations using physically positioned optical timing gates at the same locations. These measurements should ideally be collected concurrently with an independent continuous-velocity criterion, such as radar or laser, to determine whether the present findings generalize beyond a simulated GNSS context. Comparison with force-platform-derived sprint mechanics would provide an additional level of validation. Further work should also examine whether the optimal number and placement of timing gates differ across sex, competition level, sport, sprinting ability, and maximum sprint velocity.

## 5. Conclusions

The optimal timing-gate configurations were [0.75, 10, 15, 25] for comparison with the standard timing-gate configuration and [0.75, 5, 10, 30] for comparison with continuous velocity reference. These configurations were selected from those that provided acceptable estimates across all FV outcomes by prioritizing the fewest timing gates, the shortest sprint distance, and the lowest average absolute percentage difference from respective reference measure. The [0.75, 10, 15, 25] configuration required four gates over 25 m and produced an average absolute percentage difference of 0.27% with an RMSE of 1.43, whereas the [0.75, 5, 10, 30] configuration required four gates over 30 m and produced an average absolute percentage difference of 0.43% with an RMSE of 0.08. These configurations therefore provide accurate and resource-efficient alternatives to the standard protocol for national-level female rugby athletes. Alternative configurations may be preferrable when a single FV metric is the primary outcome. Further validation across populations and using physically-measured timing-gate splits is required before broader application.

## Figures and Tables

**Table 1:** Technical specifications of the GNSS units used for data collection.

Characteristics	Specification
Manufacturer and model	Apex 10 Hz, STATSports, Newry Northern Ireland
Dimensions	30 × 80 mm
Mass	48 g
GNSS sampling frequency	10 Hz
Satellite Systems	GPS, GLONASS, Galileo, and BeiDou
GNSS configuration	Augmented multi-GNSS receiver
Tri-axial accelerometer	100 Hz
Gyroscope	100 Hz
Magnetometer	10 Hz
Unit position	Upper back, between the scapulae
Variables used	Time, distance, instantaneous horizontal velocity, and instantaneous acceleration
Velocity determination	Doppler-shift solution from received GNSS satellite signals
Acceleration determination	Manufacturer-generated GNSS acceleration output; exact filtering/differentiation procedure proprietary unless confirmed by the manufacturer

**Table 2:** Percentage difference values for each respective FV variables for all 4, 5, 6, and 7 gate combinations shorter than 40 m that were not significantly different than the TG_s_-derived FV variables. The average of absolute percentage difference values (Avg %) and RMSE are also presented. The lowest percentage difference value is identified with ‘*’. The best combination (shortest distance, with fewest time gates and the lowest Avg %) is highlighted in red below.

Configuration	Gates	Total Distance	V_0_ %	F_0_ %	DRF %	Pmax %	Est40 %	RMSE	Avg %	Effect Size
0.75-10-20-25	4	25	−0.10	0.92	1.23	0.39	0.20	2.82	0.57	0.10
0.75-10-15-25	4	25	0.12	0.43	0.52	0.22	0.09	1.43	0.27	0.06
0.75-10-25-30	4	30	0.09	0.49	0.47	0.38	−0.02	2.16	0.29	0.06
0.75-10-20-30	4	30	0.41	−0.63	−0.74	−0.36	−0.07	2.29	0.44	0.10
0.75-10-15-30	4	30	0.42	−0.46	−0.67	−0.16	−0.13	1.34	0.37	0.10
0.75-15-30-35	4	35	0.78	−1.41	−1.81	−0.73	−0.20	4.87	0.99	0.24
0.75-15-25-35	4	35	0.82	−1.68	−2.02	−0.96	−0.19	6.11	1.13	0.27
0.75-10-30-35	4	35	0.14	0.35	−0.17	0.41	−0.12	2.12	0.24	0.08
0.75-10-25-35	4	35	0.25	−0.26	−0.39	−0.07	−0.11	0.71	0.22 *	0.07
0.75-10-20-35	4	35	0.51	−1.19	−1.39	−0.71	−0.14	4.43	0.79	0.24
0.75-10-15-35	4	35	0.44	−0.73	−1.00	−0.31	−0.16	2.28	0.53	0.18
0.75-5-20-35	4	35	−0.19	1.61	1.46	1.18	−0.02	6.69	0.89	0.17
0.75-5-15-35	4	35	−0.13	1.54	1.30	1.23	−0.08	6.82	0.86	0.18
0.75-10-20-25-30	5	30	0.32	−0.49	−0.54	−0.32	−0.05	1.90	0.34	0.07
0.75-10-15-25-30	5	30	0.30	−0.34	−0.45	−0.17	−0.08	1.13	0.27	0.07
0.75-10-15-20-30	5	30	0.44	−0.86	−0.98	−0.54	−0.08	3.29	0.56	0.16
0.75-10-25-30-35	5	35	0.20	−0.15	−0.26	−0.01	−0.11	0.36	0.15	0.06
0.75-10-20-30-35	5	35	0.49	−1.11	−1.31	−0.65	−0.12	4.07	0.73	0.22
0.75-10-20-25-35	5	35	0.47	−1.22	−1.36	−0.77	−0.12	4.72	0.79	0.24
0.75-10-15-30-35	5	35	0.40	−0.65	−0.89	−0.28	−0.16	2.06	0.48	0.17
0.75-10-15-25-35	5	35	0.40	−0.83	−1.01	−0.45	−0.12	2.93	0.56	0.18
0.75-10-15-20-35	5	35	0.53	−1.32	−1.52	−0.81	−0.13	4.99	0.86	0.31
0.75-5-20-25-35	5	35	−0.20	1.24	1.21	0.82	0.01	4.83	0.70	0.16
0.75-5-15-20-35	5	35	0.08	0.17	0.07	0.15	−0.05	0.84	0.11 *	0.03
0.75-5-10-20-35	5	35	−0.15	1.00	0.95	0.72	−0.03	4.13	0.57	0.15
0.75-5-10-15-35	5	35	−0.06	1.00	0.83	0.822	−0.09	4.53	0.56	0.16
0.75-10-15-20-25-30	6	30	0.36	−0.74	−0.81	−0.49	−0.07	2.93	0.49	0.14
0.75-10-15-25-30-35	6	35	0.38	−0.77	−0.95	−0.42	−0.11	2.71	0.53	0.18
0.75-5-15-20-30-35	6	35	0.09	0.22	0.09	0.21	−0.06	1.10	0.13 *	0.05
0.75-5-15-20-25-35	6	35	−0.15	1.00	0.95	0.72	−0.03	0.53	0.57	0.03
0.75-5-10-20-25-35	6	35	−0.20	0.91	0.95	0.57	0.02	3.44	0.53	0.17
0.75-5-10-15-20-35	6	35	0.00	0.26	0.22	0.19	−0.03	1.07	0.14	0.05
0.75-5-15-20-25-30-35	7	35	0.07	−0.14	0.06	0.11	−0.04	0.61	0.08 *	0.03
0.75-5-10-15-20-30-35	7	35	0.02	0.27	0.20	0.22	−0.05	1.23	0.16	0.05
0.75-5-10-15-20-25-35	7	35	−0.05	0.28	0.30	0.14	0.00	0.93	0.15	0.07

**Table 3:** Percentage difference values for each respective FV variable for all 4- and 5-gate combinations shorter than 40 m that were not significantly different from the V_cont_-derived FV variables. The average of absolute percentage difference values (Avg %) and RMSE are also presented. The lowest Avg % values are identified with ‘*’ for each gate-combination category. The best combination (shortest distance, with fewest time gates and the lowest Avg %) is highlighted in blue below.

Configuration	Gates	Total Distance	V_0_ %	F_0_ %	DRF %	Pmax %	Est40 %	RMSE	Avg %	Effect Size
0.75-5-10-30	4	30	0.91	0.01	1.44	−0.01	−0.16	0.08	0.43 *	0.06
0.75-5-15-30	4	30	1.08	−0.70	0.74	−0.56	−0.14	3.38	0.64	0.08
0.75-5-20-30	4	30	0.85	0.55	2.02	0.23	−0.04	1.82	0.74	0.07
0.75-5-20-35	4	35	0.90	−0.94	0.33	−0.75	−0.16	4.57	0.62	0.08
0.75-5-15-35	4	35	1.00	−1.44	−0.13	−1.06	−0.22	6.63	0.77	0.10
0.75-5-10-25-30	5	30	0.74	−0.07	1.55	−0.24	−0.07	1.23	0.53 *	0.08
0.75-5-20-30-35	5	35	0.78	−0.87	0.41	−0.74	−0.17	4.42	0.59	0.10

**Table 4:** For each FV metric, the ‘best’ configuration using 4, 5, 6, and 7 gates are identified comparing to both TG_s_ and V_cont_ comparative measures. The absolute error (rounded to two decimals) and sprint distance is reported each configuration.

Metric	Gates	Distance	TG_s_ Comparison	Abs Error	Distance	V_cont_ Comparison	Abs Error
P_max_	7	40	0.75-5-10-15-20-25-40	−0.24	40	0.75-5-10-25-30-35-40	−7.99
	6	40	0.75-10-25-30-25-40	0.13	40	0.75-5-10-30-35-40	−3.42
	5	35	0.75-10-25-30-35	−0.08	25	0.75-5-10-20-25	0.41
	4	35	0.75-10-25-35	−0.76	30	0.75-5-10-30	−0.11
F_0_	7	35	0.75-5-15-20-25-30-40	0.09	40	0.75-5-10-25-30-35-40	−4.16
	6	40	0.75-5-15-20-35-40	−0.09	25	0.75-5-10-15-20-25	0.98
	5	40	0.75-5-15-20-40	0.10	30	0.75-5-10-25-30	−0.41
	4	35	0.75-25-35-40	−0.24	30	0.75-5-10-30	0.04
DRF	7	40	0.75-5-15-20-30-35-40	0.00	40	0.75-5-10-15-25-30-40	0.00
	6	40	0.75-5-15-20-25-40	0.00	40	0.75-5-15-25-30-40	−0.01
	5	40	0.75-25-30-25-40	0.00	30	0.75-5-15-20-30	0.00
	4	40	0.75-25-30-40	0.00	40	0.75-5-15-40	0.00
V_0_	7	40	0.75-5-15-20-25-30-40	0.00	40	0.75-5-20-25-30-35-40	0.03
	6	35	0.75-5-10-15-20-35	0.00	40	0.75-5-25-30-35-40	0.00
	5	40	0.75-5-15-20-40	0.00	40	0.75-5-25-35-40	−0.01
	4	30	0.75-10-25-30	0.00	30	0.75-5-25-30	0.00
Est40	7	35	0.75-5-10-15-20-35-35	0.00	40	0.75-5-10-15-20-25-40	0.00
	6	40	0.75-5-15-20-25-40	0.00	30	0.75-5-15-20-25-30	0.00
	5	35	0.75-5-20-25-35	0.00	30	0.75-5-20-25-30	0.00
	4	40	0.75-5-20-40	0.00	30	0.75-5-20-30	0.00

## Data Availability

Data can be made accessible upon request.

## Supplementary Materials

The following supporting information can be downloaded at: https://www.mdpi.com/article/10.3390/s26144642/s1, Table S1: Descriptive statistical analysis of the ANOVA comparison to the baseline TG_s_ measure; Table S2: Descriptive statistical analysis of the ANOVA comparison to the baseline V_cont_ measure.
