# Supplementary material for: Does Placement of Timing Sensors and Sprinting Model Alter Force–Velocity Metrics? A GNSS Simulation Study
Source: Sensors (Basel). 2026 Jul 22;26(14):4642. doi: 10.3390/s26144642 (PMC13416514; doi:10.3390/s26144642)
Supplement: Supplementary file 1 [file sensors-26-04642-s001.zip › sensors-4414544-supplementary.pdf]

Table S1. Descriptive statistical analysis of the ANOVA comparison to the baseline TGs measure

Variable	Gates	Mauchly's test p	Greenhouse-Geisser p	Numerator df	Denominator df	F	p	Effect Size
V_0	4	0	2.53E-25	56	21000	30.248	2.49E-304	0.026
	5	0	1.21E-19	70	26250	26.544	0	0.012
	6	0	1.92E-12	56	21000	19.030	2.02E-181	0.005
	7	0	1.06E-08	28	10500	15.211	4.21E-71	0.002
	8	0	8.70E-07	8	3000	13.372	3.66E-19	7.03E-04
F_0	4	0	7.20E-35	56	21000	48.460	0	0.023
	5	0	2.46E-33	70	26250	45.965	0	0.011
	6	0	2.21E-27	56	21000	37.347	0	0.005
	7	0	6.57E-22	28	10500	31.689	1.64E-161	0.002
	8	0	2.73E-18	8	3000	28.405	5.51E-43	7.27E-04
DRF	4	0	7.78E-37	56	21000	49.487	0	0.039
	5	0	1.48E-29	70	26250	41.463	0	0.018
	6	0	4.49E-21	56	21000	31.064	3.22E-313	0.008
	7	0	1.99E-15	28	10500	25.122	1.31E-125	0.003
	8	0	3.56E-12	8	3000	21.898	1.05E-32	0.001
P_{max}	4	0	5.13E-33	56	21000	45.577	0	0.011
	5	0	1.77E-36	70	26250	48.096	0	0.005
	6	0	8.27E-34	56	21000	42.63	0	0.002
	7	0	8.69E-30	28	10500	38.252	4.86E-197	0.001
	8	0	6.18E-27	8	3000	35.521	4.13E-54	3.74E-04
Est40	4	0	7.08E-39	56	21000	56.017	0	0.011
	5	0	4.08E-21	70	26250	34.137	0	0.003
	6	0	6.63E-08	56	21000	15.289	2.29E-140	6.24E-04
	7	0	0.002	28	10500	6.807	7.67E-26	1.42E-04
	8	0	0.032	8	3000	3.927	1.25E-04	3.21E-05

Table S2. Descriptive statistical analysis of the ANOVA comparison to the baseline V_{cont} measure

Variable	Gates	Mauchly's te.	Greenhouse-Geisser p	Numerator df	Denominator df	F	fp	Effect Size
V_0	4	0	4.91E-28	56	19432	31.782	6.47E-320	0.029
	5	0	5.11E-10	70	25830	15.802	1.03E-181	0.008
	6	0	2.65E-04	56	21112	7.116	1.52E-52	0.002
	7	0	6.61E-04	28	10808	6.65	4.89E-25	0.001
	8	0	4.32E-04	8	3096	8.478	1.81E-11	0.002
F_0	4	0	8.41E-35	56	19432	41.869	0	0.024
	5	0	2.12E-22	70	25830	30.514	0	0.010
	6	0	1.22E-13	56	21112	18.152	8.44E-172	0.004
	7	0	1.61E-05	28	10808	10.037	6.05E-43	0.002
	8	0	0.017	8	3096	5.306	1.24E-06	0.002
DRF	4	0	1.09E-37	56	19432	45.931	0	0.041

	5	0	2.11E-20	70	25830	30.388	0	0.015
	6	0	1.32E-11	56	21112	17.538	4.75E-165	0.005
	7	0	5.24E-06	28	10808	8.929	5.25E-37	0.002
	8	0	0.102	8	3096	2.496	0.011	6.52E-04
P_{max}	4	0	1.32E-31	56	19432	36.378	0	0.011
	5	0	1.46E-23	70	25830	29.457	0	0.005
	6	0	5.82E-13	56	21112	18.126	1.65E-171	0.002
	7	0	2.68E-04	28	10808	9.337	3.49E-39	0.001
	8	0	0.031	8	3096	4.551	1.59E-05	0.001
Est40	4	0	6.34E-42	56	19432	54.558	0	0.012
	5	0	4.01E-14	70	25830	23.794	2.78E-291	0.002
	6	0	4.33E-04	56	21112	7.203	1.92E-53	4.59E-04
	7	0	0.093	28	10808	2.307	1.06E-04	1.00E-04
	8	0	0.445	8	3096	0.683	0.707	4.52E-05
